# Dr. George Papanicolaou: Inventor of the Pap Smear and Father of Modern Cytology

**DOI:** 10.7759/cureus.75106

**Published:** 2024-12-04

**Authors:** Abigail Dugas, Nadiya A Persaud, Latha Ganti

**Affiliations:** 1 Research, Orlando College of Osteopathic Medicine, Winter Garden, USA; 2 Medical Science, The Warren Alpert Medical School of Brown University, Providence, USA

**Keywords:** biographies, cytology, historical vignette, medical innovation, medical stories, pap test

## Abstract

The Pap smear is widely recognized in medicine as the single most successful contributor to cancer screening and preventative care. Women have Dr. George Papanicolaou (1870-1962) to thank for this groundbreaking contribution to their healthcare-a discovery that, fascinatingly, was made incidentally during his study of ovulation cycles in guinea pigs. He found that vaginal smears from his subjects with cervical cancer, or those who later developed the disease, yielded abnormal cells that were notably discernible under a microscope when compared to healthy cells. Dr. Papanicolaou, both a physician and scientist, was a devoted researcher who conducted revolutionary studies alongside his wife at Cornell University for nearly 50 years. This paper will highlight the course of Dr. Papanicolau’s life, how he found himself searching for his niche in the world of medicine and then scientific research, leading to his incidental discovery of the efficacy of the Pap smear in cancer screening and preventative measures. Dr. Papanicolau’s legacy lives on through the contributions he made to the intersection of women’s health with cancer prevention, and his devotion to his research continues to serve the field of medicine, now 50 years after his death.

## Introduction and background

**Figure 1:**
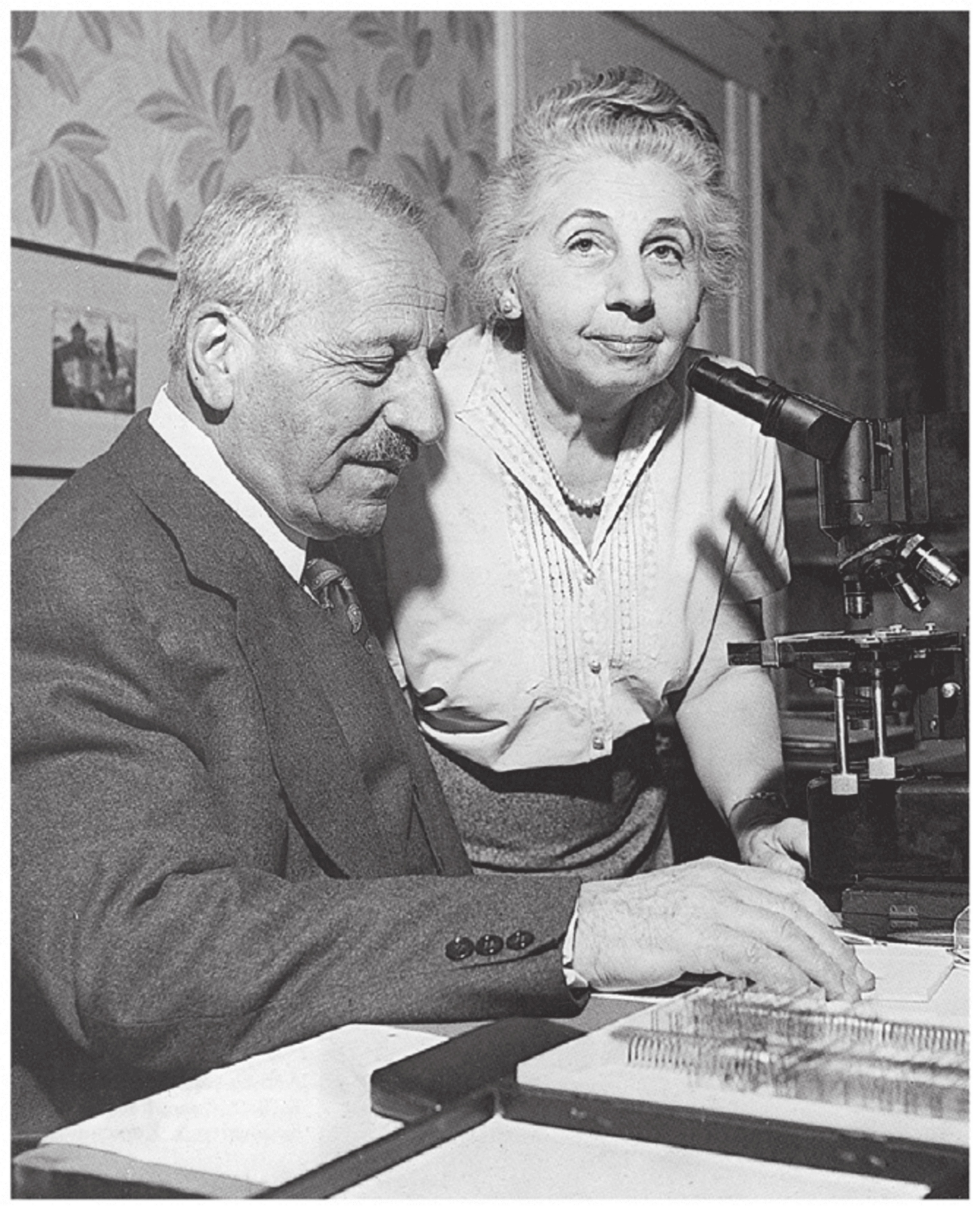
Dr. George Papanicolaou with his wife, Mary Papanicolaou. Image available via license: Creative Commons Attribution-NonCommercial-NoDerivatives 4.0 International (https://creativecommons.org/licenses/by-nc-nd/4.0/) [7]

Dr. Papanicolaou was born on May 13, 1883, in Kimi, Greece, as the third of four children to Maria G. Kritsouta and Nicholas A. Papanicolaou [1,2]. His father, a well-known physician, senator, and mayor in their town, wanted George to follow in his footsteps and pursue a medical career [3]. However, George initially rejected this idea and first attended the University of Athens, where he earned a degree in humanities and studied music as a violinist [4,5]. Later, he returned to the University of Athens to fulfill his father’s wishes to attend medical school, graduating with top honors in 1904 [2,4,6]. After graduating from medical school, he briefly worked as an assistant surgeon in the military and as a practitioner for leprosy patients. However, he soon realized that he did not enjoy practicing medicine and decided to shift his focus to research. This decision prompted him to pursue a PhD in zoology, which he earned in 1910 from the University of Munich. During his doctoral studies, he worked under Dr. Ernst Haeckel, one of the earliest supporters of Darwinism, and Professor August Weismann, an early geneticist [1,3]. Shortly after earning his PhD, he met and married his wife, Mary (Figure 1) [3]. Due to the limited academic opportunities in Athens, Dr. Papanicolaou explored other endeavors in Europe before he and his wife chose to emigrate to the United States in search of greater research opportunities [5]. Throughout the nearly 50 years of dedication to his work, Mary supported Dr. Papanicolaou in his research, even serving as his test subject and becoming one of the first individuals to receive a Pap smear.

## Review

Early career

After completing medical school, Dr. Papanicolaou embarked upon his medical career as an assistant surgeon in the military [4]. He later returned home to care for leprosy patients for two years [4,6]. However, much to his father’s disappointment, he found little enjoyment in practicing medicine and decided to shift his focus to research in the biological sciences [4]. In 1910, he earned a PhD in zoology from the University of Munich in Germany, where he published a thesis on the sex differentiation of daphnia [1,6]. Struggling to find biological research opportunities in Greece, he ventured to Monaco, where he spent one year conducting research on the cell functions of marine organisms at the Oceanographic Institute of Monaco [2]. While there, he joined a marine expedition led by Prince Albert of Monaco, serving as the physician on board [2,5]. At the start of the Balkan War in 1912, he returned to military service and was eventually promoted to second lieutenant in the medical corps [2]. During this time, he learned about research opportunities in the United States from American volunteers, inspiring his later ventures [1,6]. With very little money, he and his wife moved to the United States in 1913 [3].

Moving to America 

When the Papanicolaous arrived in the United States, they took on various jobs. Mary worked as a seamstress, while George worked as a violinist in a restaurant and a newspaper clerk and even dabbled as a rug salesman for a day [1,4,6]. Dr. Thomas Hunt Morgan of Columbia University took great interest in Dr. Papanicolaou’s research, later helping him secure a part-time position in the Department of Pathology at New York University. About a year later, he assumed the role of a full-time researcher in the Department of Anatomy at Cornell University Medical College, working under Professor Charles Stockard [3,6]. His wife, Mary, also secured a position at Cornell University to assist him with his research ventures [3]. Dr. Papanicolaou served as a tenured researcher at Cornell University for 47 years, where he established the Papanicolaou Cytology Laboratory [5].

Early research

Dr. Papanicolaou's research initially focused on gender differentiation in guinea pigs, specifically via X and Y chromosomes in sperm and ova [3]. While laying the groundwork for the Pap smear, he studied the ovulation cycle in guinea pigs, seeking to pinpoint the timing of ovulation [4]. He accomplished this by collecting vaginal secretions and cells from the guinea pigs and studying them under a microscope [3]. He found that the epithelial cells of the vagina presented in various forms, cycling every 15 to 16 days, and established a correlation between this cycle and changes occurring in the reproductive organs [1,3]. In 1920, he shifted his focus on cytology research to the human reproductive system [2].

The discovery of the Pap smear 

Dr. Papanicolaou concentrated on studying the various cytological changes in the vaginal epithelium and their relationship to ovulation cycles. He collected data by performing vaginal smears on his subjects, using a speculum to visualize and collect samples from the cervix [3]. Incidentally, he found that cancerous cells appeared abnormal with distinctively appearing nuclei [1]. He started by collecting samples from women at the New York Women's Hospital in New York City, where he confirmed that his technique could successfully detect malignant cells from just a smear and a swab [2]. He presented this technique for the very first time in 1928 at the Third Race Betterment Conference in Battle Creek, Michigan [1-3]. His discovery was initially met with skepticism, as his colleagues were hesitant to believe that a confirmed diagnosis of cancer could be made without a biopsy. For several years, he neglected this finding, until he began working with Dr. Herbert Traut, a gynecological pathologist. Dr. Traut recognized the importance of Papanicolaou’s technique and helped him to gather more data to demonstrate its effectiveness [1-3]. Together, the two conducted a clinical trial at New York Hospital, ensuring that every woman attending a gynecology visit underwent a Pap smear. Through this trial, Dr. Papanicolaou identified several cases of cervical and uterine cancer that would have otherwise gone undiagnosed. Some of these tumors were asymptomatic and not visible to the naked eye. Without Dr. Papanicolaou's innovative Pap smear test, they likely would have progressed beyond the point of treatability before being detected [2]. The findings from this trial were published in 1941: “The diagnostic value of vaginal smears in carcinoma of the uterus” [8]. Two years later, in 1943, he published his findings of over 3,000 cases in an illustrated monograph: “Diagnoses of uterine cancer by the vaginal smear” [4]. These works demonstrated the preparation of vaginal and cervical smears, which showed a variety of cytologic changes in the menstrual cycle and of various pathologies of the vagina and cervix, especially that of cervical or endometrial cancer [1]. Over time, Dr. Papanicolaou’s continued studies further proved the efficacy of his technique, and he worked to train others to perform and read the smears, allowing them to be adopted into standard practice [4]. The Pap smear became standard in women’s healthcare settings by the late 1940s and greatly decreased the incidence of deaths due to cervical cancer [4].

Beyond the Pap smear

Following the success of the Pap smear, Dr. Papanicolaou continued as a Professor of Anatomy and continued to explore his passion for cytology research [1]. In 1954, Dr. Papanicolaou published the Atlas of Exfoliative Cytology, a comprehensive review of his research highlighting the cytological differences between healthy and diseased tissues [1,4]. This atlas was revolutionary in developing the modern medical specialty of cytopathology [4]. After retiring from Cornell University in 1957, Dr. Papanicolaou attended the first International Cytology Conference in Brussels with his wife [1]. During his time in Europe, he made a brief return to his homeland, Greece, aspiring to open a cancer research institute there, but the effort was unsuccessful [5]. Instead, in 1961, he relocated to Miami to lead the newly established Cancer Institute of Miami [1]. Sadly, Dr. Papanicolaou passed away from a myocardial infarction in 1962, just three months after he and his wife arrived in Miami [1]. In November of 1962, the Cancer Institute of Miami was renamed the Papanicolaou Cancer Research Institute in his honor [1,2,5].

Legacy and recognitions 

**Figure 2:**
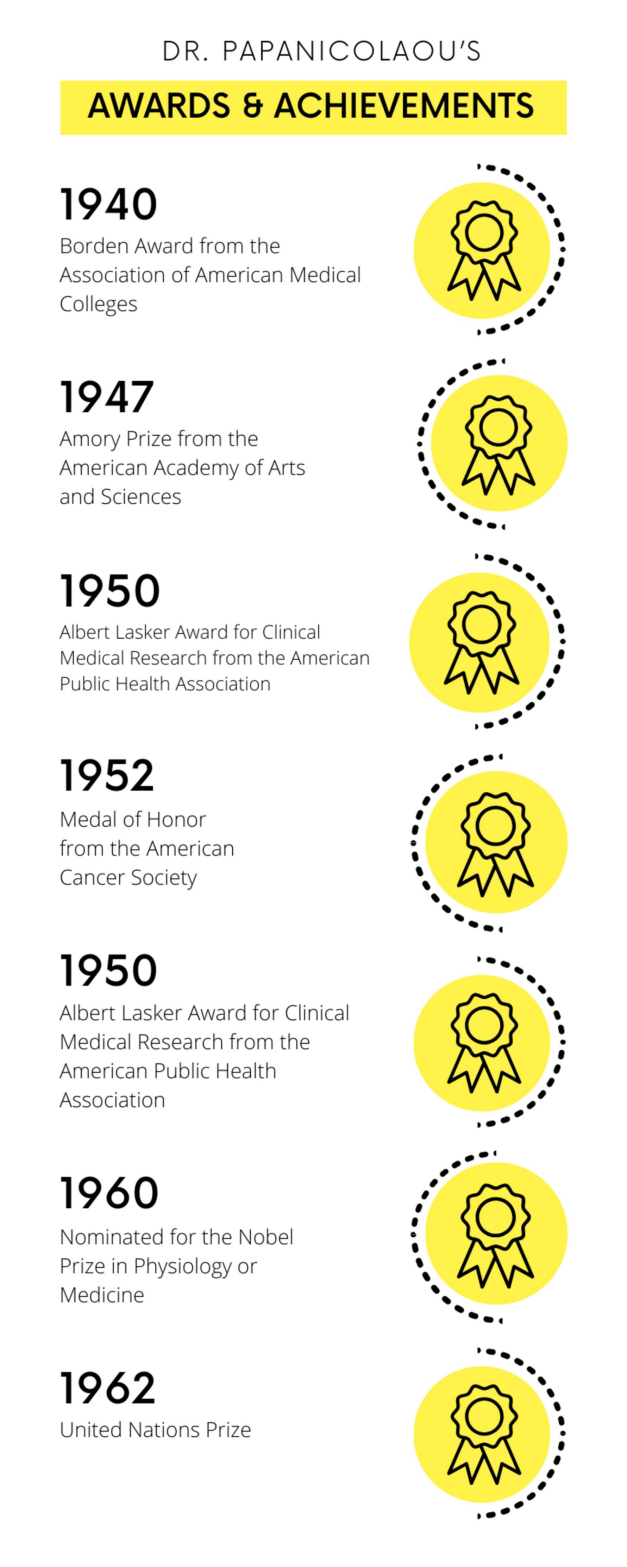
Dr. Papanicolaou's awards & achievements Designed by Nadiya A. Persaud on Canva.com

Although the discovery of the Pap smear came relatively early in his career, Dr. Papanicolaou remained immensely committed to his research throughout nearly 50 years of work. He devoted himself to his work seven days a week, never taking a vacation [4]. Throughout his life, he authored four books and published over 150 scientific articles [3,4]. Dr. Papanicolaou's contributions to the medical field are widely recognized through numerous awards and honors, including the Borden Award from the Association of American Medical Colleges in 1940, the Amory Prize from the American Academy of Arts and Sciences in 1947, the Albert Lasker Award for Clinical Medical Research from the American Public Health Association in 1950, and the Medal of Honor from the American Cancer Society in 1952 [4]. He also earned honorary membership in prestigious organizations such as the Academy of Athens, the Obstetrical and Gynecological Society of Athens, and the New York Academy of Sciences [4]. Additionally, he was nominated for the Nobel Prize in Physiology or Medicine in 1960 and received the United Nations Prize in 1962 (Figure 2) [1,4]. His likeness has even been featured on the Greek drachma currency and a postage stamp in 1978 by the US Postal Service [2,6].

## Conclusions

Dr. Papanicolaou's enduring legacy echoes throughout various medical specialties, including cytopathology, gynecology, and oncology. Prior to the discovery of the Pap smear, cervical cancers were often diagnosed only after symptoms presented, typically when the disease had progressed too far for effective treatment. The Pap smear revolutionized cancer detection by providing an efficient and relatively inexpensive method to detect cervical and uterine cancers early, significantly improving survival rates. The Pap smear is widely recognized as one of the most successful cancer screening tools, and Dr. Papanicolaou's extensive research and publications in cytopathology laid the foundation for this crucial medical specialty. His unwavering dedication to research led to groundbreaking discoveries that continue to be invaluable to modern medicine, and his life and legacy will always be remembered.
